# Artificial Neural Network classification of operator workload with an assessment of time variation and noise-enhancement to increase performance

**DOI:** 10.3389/fnins.2014.00372

**Published:** 2014-12-01

**Authors:** Alexander J. Casson

**Affiliations:** ^1^Sensing, Imaging and Signal Processing Group, School of Electrical and Electronic Engineering, The University of ManchesterManchester, UK; ^2^This Work was Carried Out While the Author was with the Optical and Semiconductor Devices Group, Department of Electrical and Electronic Engineering, Imperial College LondonLondon, UK

**Keywords:** Artificial Neural Network, Augmented Cognition, EEG, noise-enhanced processing, passive BCI, stochastic resonance, workload classification

## Abstract

Workload classification—the determination of whether a human operator is in a high or low workload state to allow their working environment to be optimized—is an emerging application of passive Brain-Computer Interface (BCI) systems. Practical systems must not only accurately detect the current workload state, but also have good temporal performance: requiring little time to set up and train the classifier, and ensuring that the reported performance level is consistent and predictable over time. This paper investigates the temporal performance of an Artificial Neural Network based classification system. For networks trained on little EEG data good classification accuracies (86%) are achieved over very short time frames, but substantial decreases in accuracy are found as the time gap between the network training and the actual use is increased. Noise-enhanced processing, where artificially generated noise is deliberately added to the testing signals, is investigated as a potential technique to mitigate this degradation without requiring the network to be re-trained using more data. Small stochastic resonance effects are demonstrated whereby the classification process gets better in the presence of more noise. The effect is small and does not eliminate the need for re-training, but it is consistent, and this is the first demonstration of such effects for non-evoked/free-running EEG signals suitable for passive BCI.

## 1. Introduction

Augmented Cognition is a recent research concept focusing on creating the next generation of Human-Computer Interaction devices (Schmorrow and Stanney, 2008). Closed-loop Brain Computer Interfaces (BCIs) are a classic example of such systems. In these, a human uses a computer whilst simultaneously the computer monitors the human and changes its operation based upon the results. Changes might be made to the outputs presented, to the input streams which are used, or to the levels of automation and assistance that are provided, amongst others. For example, workload monitoring systems aim to detect when an operator is in a high or a low workload state to potentially change the speed at which information is presented. As such the work flow and operating environment can be optimized in a real-time and time-varying manner (Wilson and Russell, 2007). Alternatively, workload monitoring could be used to enhance human training: the mental load of a new task can be objectively measured and training times increased or decreased to terminate the training only when the task involves a low level of effort (Ayaz et al., 2012).

Emerging passive BCIs use the spontaneously produced *free-running* EEG (electroencephalogram) signals that are naturally present on the scalp due to the normal functioning of the brain without any specific stimuli present or the user consciously controlling their brain activity (Zander and Kothe, 2011). Workload classification is quickly emerging as a *killer app* for passive BCI as it is particularly suited to being based upon free-running EEG as opposed to evoked responses. At the extreme fatigue end: sleep onset is characterized in free-running EEG by a reduction in alpha (7–12 Hz) activity, with this being replaced by theta (4–7 Hz) activity (Rechtschaffen and Kales, 1968). This has long been used clinically for sleep staging and there are numerous recent papers (see for example Christensen et al., 2012; Dijksterhuis et al., 2013) demonstrating that frequency band changes can be used to identify less extreme changes in vigilance level.

As with many BCI systems the challenge for workload monitors now is in moving out-of-the-lab and into uncontrolled environments which are significantly corrupted by noise, and in creating systems that are reliable, robust and re-usable. For machine learning based systems an essential parameter in this is the training required to set up the classification process. During training, EEG data which is known to arise during a particular workload state is presented to the classifier, which it then uses to *learn* classification boundaries which can be applied to new EEG data where the workload state is not known. Ideally this classifier generation and training process:

1. Leads to the best classification performance when applied to new EEG data.
2. Requires little training data.
3. Maximizes the time before re-training is required.

Traditionally much focus has been attached to maximizing condition 1, ensuring that the classification system has good generalisability and can be correctly applied to previously unseen data. However, for practical systems this is not the only objective, and conditions 2 and 3 which determine the *temporal performance* of the system are of critical importance. Recent work (Estepp et al., 2011; Christensen et al., 2012) has suggested that the performance of workload monitors is not constant, instead degrading as the time gap from the training session is increased. (Christensen et al., 2012) showed that some of this performance loss could be recovered by increasing the amount of training data used and including data from multiple EEG sessions: each new EEG session would start with a short new training period to supplement the existing training data that was collected in previous sessions, potentially some time ago.

However, each time the classifier has to be re-trained the user must be placed into a known high or low workload state and new EEG data collected. This requires both a considerable amount of effort, and decreases the time during which the system can be practically used to perform useful classifications. It is thus essential to devise and investigate new techniques that can potentially be used to improve temporal performance without requiring more training sessions to be carried out, and this paper begins this investigation.

This paper presents an Artificial Neural Network based workload classifier for determining operator state from the EEG (Section 2) with its performance evaluated in two novel ways. Firstly, focus is given to the temporal performance of the classification process, quantifying how long a system developed using very little training data can be used before re-training is required (as opposed to maximizing the general performance, condition 1 above). Whilst very good short term performance is obtained, it is verified that the performance does degrade with time.

Secondly, *noise-enhanced processing* is investigated as a technique to mitigate the change in performance. In this, artificially generated corrupting noise is deliberately added to the otherwise raw EEG input signal to evoke *stochastic resonance* from the classification process. This is an effect in non-linear processes whereby performance actually gets better in the presence of small amounts of noise (Kay, 2000; Chen et al., 2007). The method is particularly interesting to explore as noise is widespread in ultra-portable out-of-the-lab EEG recordings, and creating algorithms that are firstly robust to the presence of noise and then even enhanced by the presence of noise can completely change how ultra-portable EEG systems are designed.

**Figure 1:**
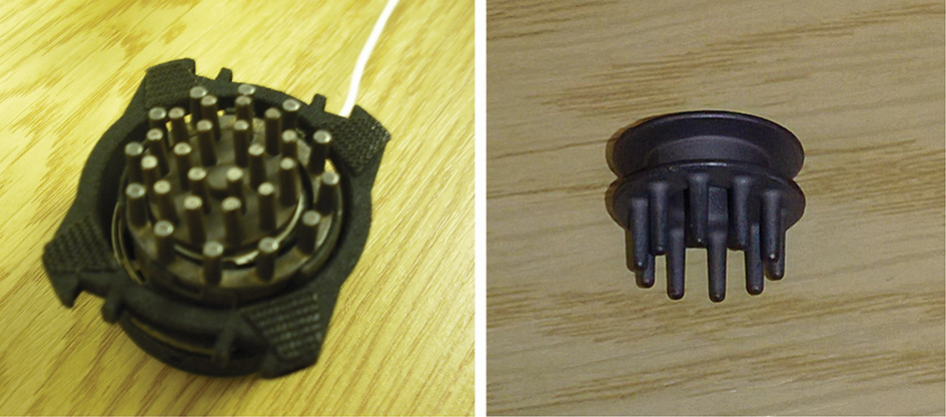
**Two state-of-the-art commercially available dry EEG electrodes show the common *fingered* configuration**. Photographs by the author.

As an illustration, nearly all modern dry EEG electrodes are based upon having *fingers*, rather than discs, for easier penetration through the hair, as illustrated in Figure 1. However, electrode contact noise is a function of the electrode area (Huigen et al., 2002), and electrode fingering decreases this area. In-depth measurements of dry electrode performance have been presented (Chi et al., 2010; Gandhi et al., 2011; Slater et al., 2012) but most studies report only a correlation coefficient between EEG recorded at nearby locations with wet and dry electrodes. Typical values reported are: >0.93 (Xu et al., 2011); 0.89 (Matthews et al., 2007); 0.83 (Gargiulo et al., 2008); 0.81–0.98 (IMEC, 2012); 0.68–0.90 (Patki et al., 2012); 0.39–0.85 (Estepp et al., 2009). New results in this paper show that even with up to 15 μVrms of artificial noise added to the raw EEG traces, correlations in-line with those reported for dry electrodes are found.

Further, in general electronics there is often a trade-off between power consumption and the effective noise present (Harrison and Charles, 2003; Xu et al., 2011; Cuadras et al., 2012). A number of high quality, highly miniaturized wearable EEG units have become commercially in recent years, but with battery lives typically in the 8 h range (Casson, 2013). This power performance still falls far short of *pick up and use* devices and substantial improvements in system power consumptions are still required to realize units that can be trusted to be re-usable session after session. If BCI applications can be enhanced by noise the design of ultra-portable EEG systems is completely changed: more noise is now desirable and this potentially allows the use of even smaller electrodes and lower power consumption processing electronics.

A preliminary investigation of using noise-enhancement in workload monitoring was presented in Casson (2013). However, this was restricted to the investigation of two subjects and the use of a single Artificial Neural Network based algorithm. The results here have been extended to eight subjects and include the use of an array of neural networks to enable collective decision making.

## 2. Materials and methods

### 2.1. Workload task and data

In this work scalp EEG data is used to classify an operator as being in either a high or low workload state whilst they are performing a flight simulator task (Comstock and Arnegard, 1992; Miller Jr., 2010). This task was designed to represent aircraft operations, and particularly those of remote piloting. The data was recorded as part of the 2011 Cognitive State Assessment Competition (Estepp et al., 2011; Christensen et al., 2012) and is publicly available from the competition organizers.^1^ It consisted of two EOG channels (vertical and horizontal) and 19 EEG channels in standard 10–20 locations (Fp1, Fp2, F7, F3, Fz, F4, F8, T3, T5, C3, Cz, C4, T4, T6, P3, Pz, P4, O1, O2). All channels used a mastoid reference and ground and a 256 Hz sampling rate.

A total of 118 EEG recordings were performed using eight subjects (15 tests in six subjects, 13 tests in two) on five separate days spread over a month. Each recording day consisted of three 15 min EEG sessions, allowing the temporal performance of the workload monitor to be evaluated on a number of scales. Firstly, within each each 15 min session the EEG data can be divided into training and testing periods which are separated in time by seconds or minutes. Recordings on the same day are separated by minutes or hours, while recordings on different days are separated by days and weeks, and up to a month.

The simulator task was set up such that the difficulty varied dynamically in order to induce known high and low workload states in the operator. No measure of task error was present in the publicly available data and instead the user workload is inferred from the set simulator difficulty. A total of 5 min was spent in each state, with at least a 1 min transition present between task segments classed as high and low workload. Here, only the high and low workload monitoring data segments are analyzed, with the transition segments being discarded. All subjects were trained in the operation of the simulator before the workload monitoring experiment was carried out, eliminating learning effects in the operators themselves.

### 2.2. Classification engine

The workload classifier is a feedforward backpropogation Artificial Neural Network (ANN) with five hidden layers (Duda et al., 2001). The network is trained on EEG frequency domain information from seven frequency bands: 0–4 Hz, 4–7 Hz, 7–12 Hz, 12–30 Hz, 30–42 Hz, 42–84 Hz, 84–128 Hz; calculated using a 1024 point FFT. This gives a total of 147 input features, and these are generated in 30 s epochs with 25 s overlap such that the assessment of operator state is updated every 5 s. All features are zero mean and unit standard deviation normalized before being passed to the network for classification. The network training used the scaled conjugate gradient backpropagation method and incorporated an early stopping training/validation/testing data split to avoid overfitting. Only 50% of the training vectors in each block of training data were used directly for network training. During network development 10 different networks with random starting weights/biases were used with different random selections of the feature vectors placed in the training sets.

After the classification has been performed, the output of the ANN is a binary state placing the subject into one of the workload categories, with this assigned as the class with the maximum output from the ANN. The classification performance is reported as the percentage of epochs in each testing set which are correctly matched with the known high or low workload in that period in the simulation.

### 2.3. Average and temporal performance assessment process

Multiple trained ANNs are generated and evaluated here to assess the performance of the classification process in three ways: the time independent average performance, the temporal performance, and the temporal performance when noise enhancement techniques are applied.

The time independent average performance is found by using a leave-one-out cross validation procedure where all but one of the EEG recordings are used to train the ANN, and the remaining EEG record is used to test the ANN. This process is done on a per-subject basis, and is repeated using all of the different EEG records as the test data. The output is multiple out of sample test performances which are averaged to obtain an overall figure. This is a standard ANN development procedure (Duda et al., 2001), but it uses large amounts of training data (approximately 150 min per subject) and it does not maintain the temporal ordering of the EEG recordings. In many instances the training data will come from time points after the test data (the procedure is non-causal) and the overall average figure does not reflect changes in ANN performance over time.

**Figure 2:**
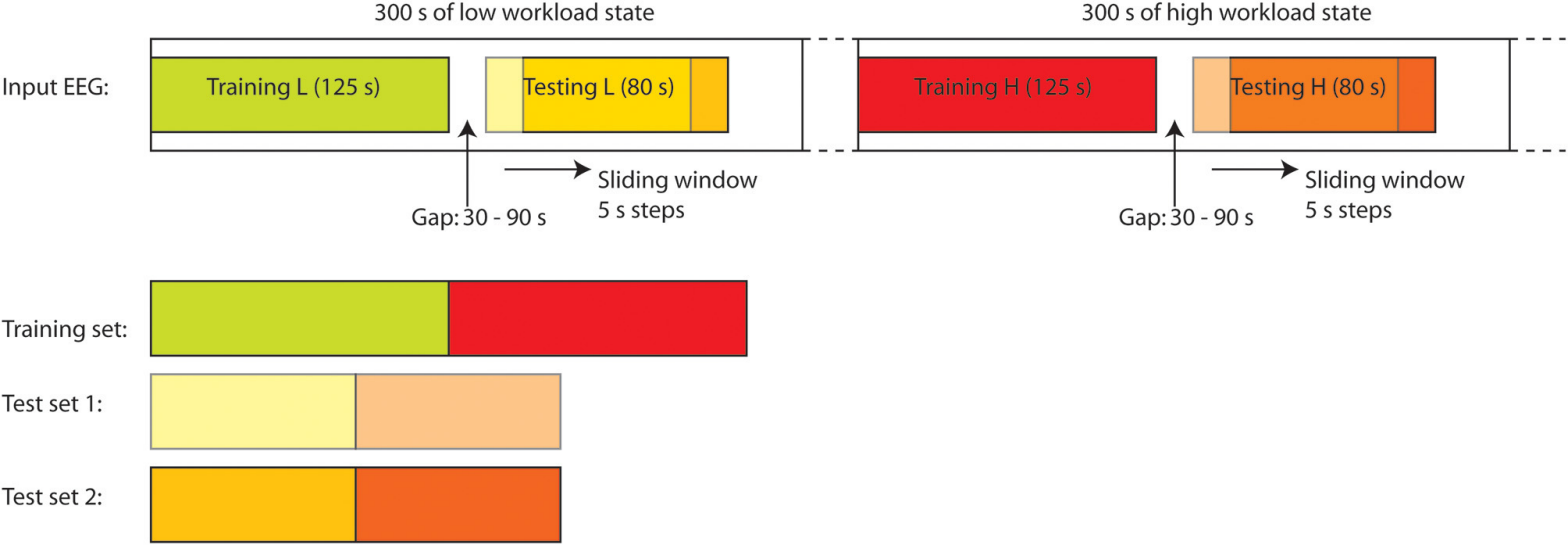
**Training/validation and test data sets are generated from a sliding window allowing the time distance from the training data to be varied and performance evaluated as a function of this distance**.

To assess the temporal performance a modified procedure is used, which maintains causality and uses much less training data. Firstly, a unique ANN is generated for each of the 118 EEG sessions using training data from the start of an EEG session. This network is used to monitor the operator workload in the remainder of this session and demonstrates the *same session* performance when the training and test data segments are very close together in time. The procedure for sub-dividing each EEG session into training/validation data and prospective testing data is illustrated in Figure 2. Here, the first 20 epochs (125 s duration) from each of the high and low workload periods are used for generating the network. The remainder of the data is used for testing. For the performance evaluation 11 epochs (80 s) of test data are assessed at a time: this vector is then stepped through all of the available test data epochs to show the achieved classification performance as a function of time. These values can then be averaged to produce an overall figure which does reflect changes in ANN performance over time.

The *cross session* temporal performance is then evaluated by re-using the ANNs trained as above (from a part of one session only) and testing them using EEG data collected in different recording sessions. In this case the test EEG is not split into training/test segments, and is instead all used as test data. Multiple time scales are investigated using different configurations of the available data:

- Time-scale of minutes: the ANN trained on the first recording session on a day is tested using data from the second recording session on that day. This is repeated for all 5 days of recording.
- Time-scale of hours: the ANN trained on the first recording session on a day is tested using data from the last recording session on that day. This is repeated for all 5 days of recording.
- Time-scale of days: the ANN trained on the very first recording session is tested using data from all of the other EEG recording sessions no matter when they were performed, some up to a month later.

All of these networks are kept subject specific, and it can be seen that the testing is purely prospective: data from different sessions is not mixed, testing data can only be from the future compared to training data, and the objective is to use little training data to accurately classify operator states which are significantly removed in time from the training data.

### 2.4. Noise-enhanced performance assessment process

To investigate noise-enhanced processing two analyses are carried out here. Firstly, to determine suitable noise levels to inject, a raw recorded EEG trace is compared to the same EEG trace after it has had artificial white Gaussian noise deliberately added to it. The correlation coefficient is then found, allowing a comparison with the correlations found in typical wet vs. dry EEG electrode studies (see discussion in Section 1). The white Gaussian noise is generated in Matlab using the wgn function with independent noise streams added to each of the EEG channels. For this analysis a single complete 12.5 h EEG recording is used; the previously publicly available data from (De Clercq et al., 2006; Vergult et al., 2007). This long EEG record is split into multiple shorter duration EEG sections, and the correlation in each section plotted against the duration of the section. This allows the maximum, minimum and median correlation coefficients over time to be found.

**Figure 3:**
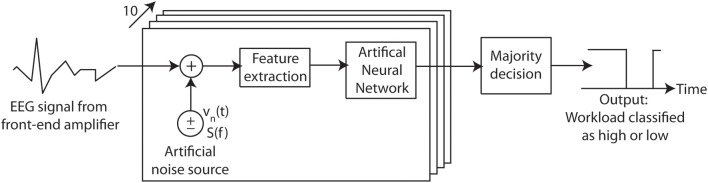
**Input referred additive noise is used to artificially corrupt an EEG recording before it is passed to an array of workload classification Artificial Neural Networks**. By varying the Root-Mean-Squared (RMS) amplitude of the noise introduced the effect on the algorithm detection performance can be investigated. Here the artificial noise source is white, has an instantaneous value *v*_*n*_(*t*), power spectral density *S*(*f*) and bandwidth of 128 Hz.

Secondly, to assess the noise-enhancement, there are numerous different ways in which noise corrupted EEG data can be passed to the ANN in order to optimally evoke stochastic resonance from the classification procedure, and only one approach is evaluated here. In this, illustrated in Figure 3, 10 identical ANNs are used in parallel each driven by EEG traces which have been corrupted by independent noise sources. The classification process is thus repeated 10 times with the output class being decided by a majority vote (in the case of a tie the output is put into the high workload state). The use of artificial noise thus allows multiple attempts of the classification for each individual EEG epoch, which would normally only be possible once.

This novel *testing with noise* is employed here to assess the cross session performance. *Training with noise* is a common technique used to increase the accuracy of Artificial Neural Networks by adding small levels of noise to the training data before training the network (Duda et al., 2001). (The aim is to do this multiple times and make the available training data more variable and more representative of future unknown data.) However, this is not employed here. The ANNs are created and trained as detailed above for assessing temporal performance, using the raw recorded EEG signals to generate the input features. Artificial noise is only added in during the testing process. To demonstrate that the results are repeatable, multiple-runs of this 10 ANN configuration using independently generated noise cases have been carried out.

## 3. Results

### 3.1. Average and temporal performance

Across all subjects the average performance from the leave-one-out cross validation procedure is 73%. The per subject performances are given in Table 1. This is a satisfactory level showing that the networks can be used for determining the operator state, and this information potentially fed back in order to optimize operating procedures.

**Table 1:** **Classification performance when using the leave-one-out cross validation to assess average performance**.

**Subject**	**Performance(%)**
A	68.4
B	73.5
C	72.6
D	62.8
E	79.8
F	75.5
G	68.4
H	79.6

**Figure 4:**
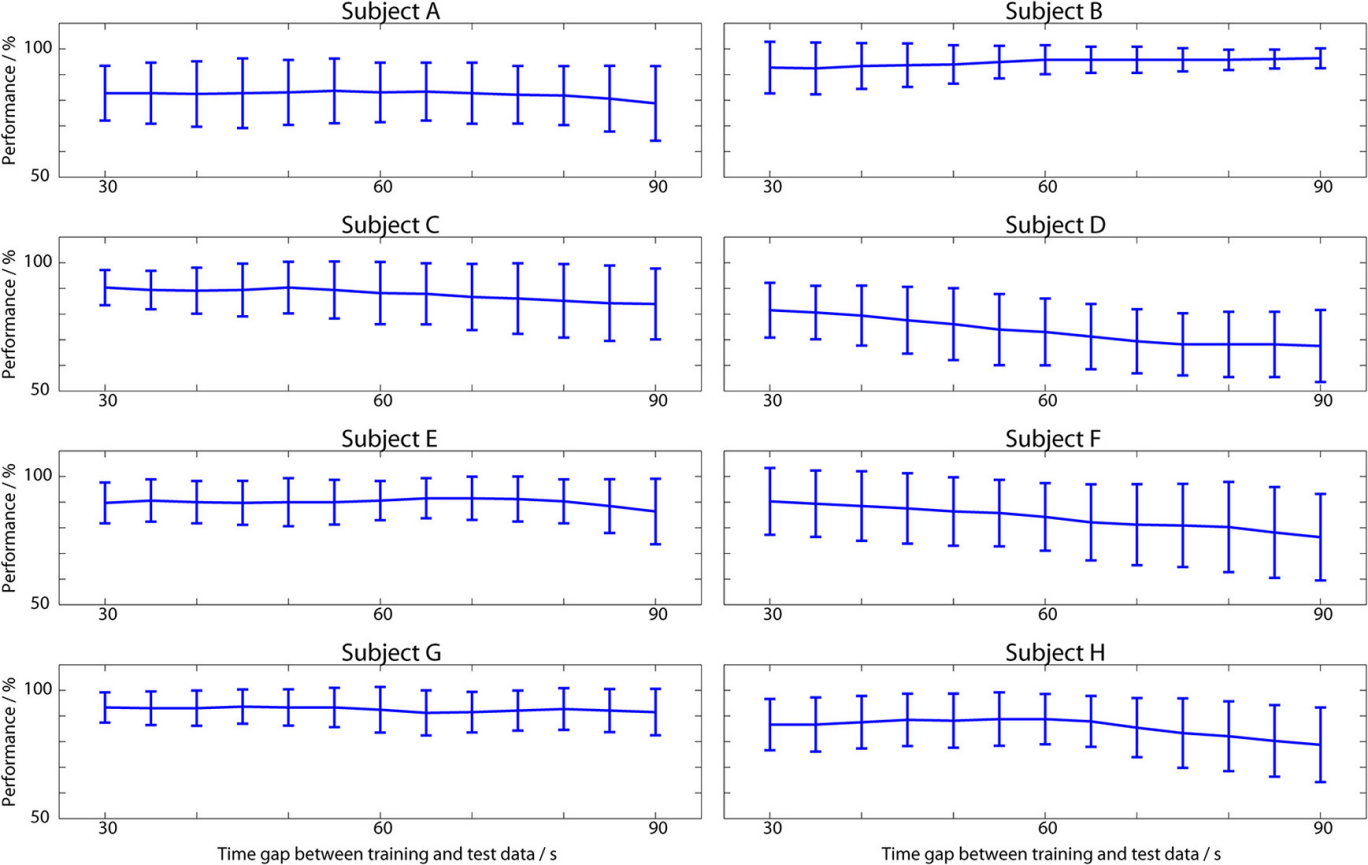
**Classifier performance when test data is from the same EEG session as the training data**. Plotted lines show the mean performance across all 15 sessions in each subject (13 for C and D) and the mean absolute deviation from this.

The *same session* temporal performance is shown in Figure 4. For illustrating the spread of results, Figure 4 breaks down the performance per subject, and plots the mean performance as the time distance between the training and test data is increased. Vertical lines illustrate the distribution of results across the 15 EEG records from each subject (13 in subjects C and D). Combining all of these results together the overall average performance, where all the performances in Figure 4 are averaged to produce a single value for each person and then averaged again across the 8 subjects, is 86%.

This is higher than the 73% from the cross-validation results because the ANNs have been trained on only a small amount of data and the ANNs generalize well to new data close in time to this, at the cost of worse performance as the time gap increases. From Figure 4 it is apparent that even over the time span of seconds to minutes the performance is not constant, with noticeable variations present. In subject B the average classification performance gets better over time, but for all of the other subjects there is a decrease in the average performance as the training data becomes increasingly distant in time, with a mean correlation of −0.77.

**Figure 5:**
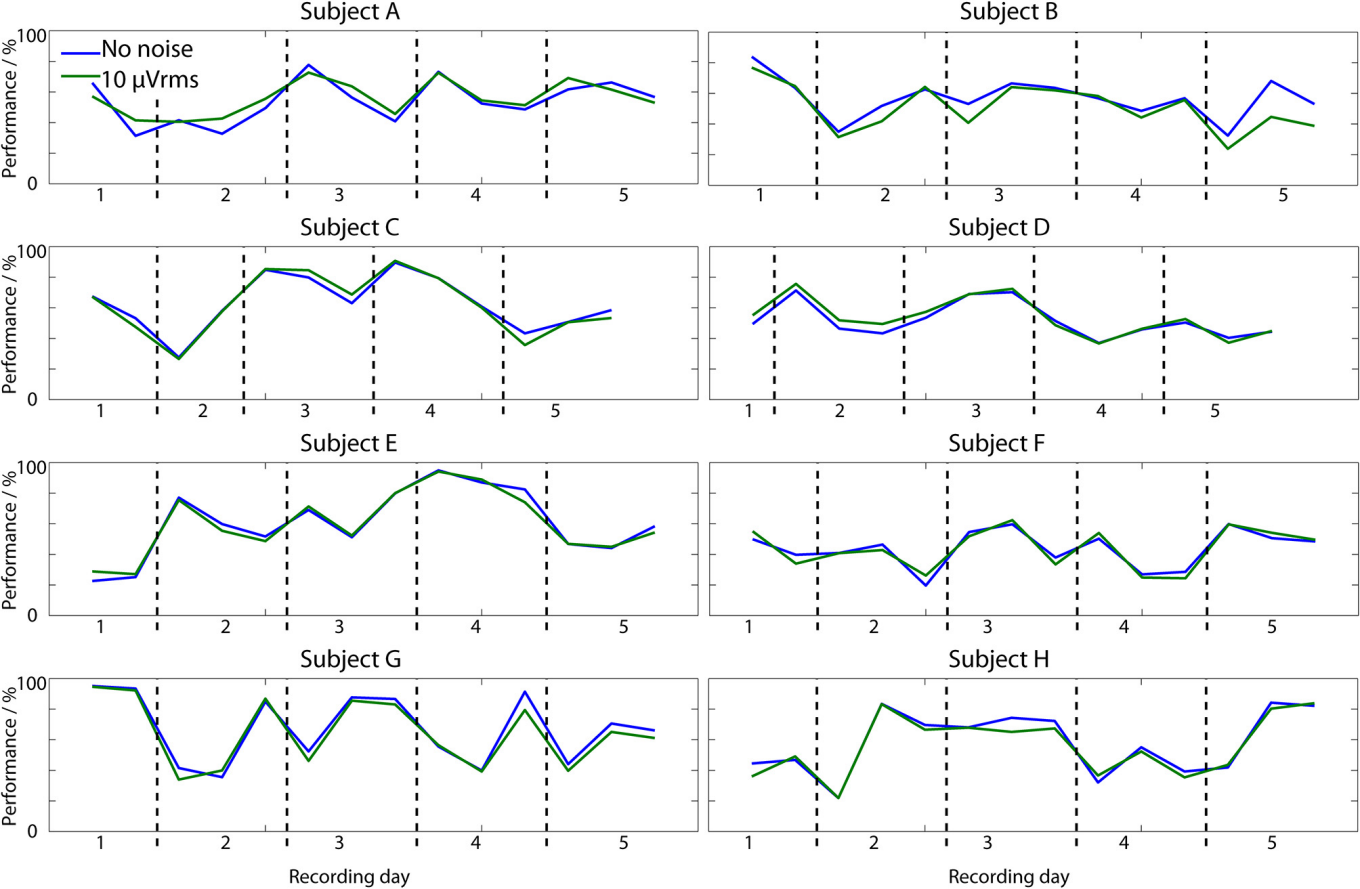
**Workload classification performance in 14 EEG sessions (12 in subjects C and D) when the ANN is trained on 250 s of data from the first recording session and tested on all of the other EEG records, up to a month apart from the training session**. Results are shown for both the no noise classifier and the ANN array with 10 μVrms of added noise.

The impact of extending the time gap to days is shown in the *cross session* results in Figure 5 which illustrates how well the ANN generated in the very first EEG session can be directly re-used over a long time span. The overall average performance is 57%. To compare this to chance a re-sampling approach has been used where the state classification from the ANN is replaced with that from a random number generator. Random values are drawn from a uniform distribution with it being equally likely to mark an EEG epoch as high or low workload. This artificially generated classification output is then analyzed in exactly the same way as the true ANN output. Simulations over 1000 runs show that the mean performance of this random classifier is 50.0%, and the best chance performance is 52.1%. This indicates that the 57% performance of the ANN is above the chance level, although it is unlikely to be of practical use.

Nevertheless, within this 57% wide per-session performance variances are seen. Many of the records achieve very good classification performances above 70%, even many days after the training session. Similarly, however, many perform at the chance level, and indeed some perform substantially worse than chance such that better classification would be actually obtained by inverting the output of the classification process. From this, it is clear that in some cases it is possible to directly re-use the workload classifier across multiple days, but this must be coupled with a method for assessing whether good classification performance is likely to be obtained.

### 3.2. Noise-enhanced performance

Figure 5 also shows the *cross session* performance of the ANN array when used with 10 μVrms of artificially generated noise. This result is noise-robust: in the 10 μVrms case the average performance is decreased only to 56%, while with 5 μVrms of added noise it remained at 57%.

**Figure 6:**
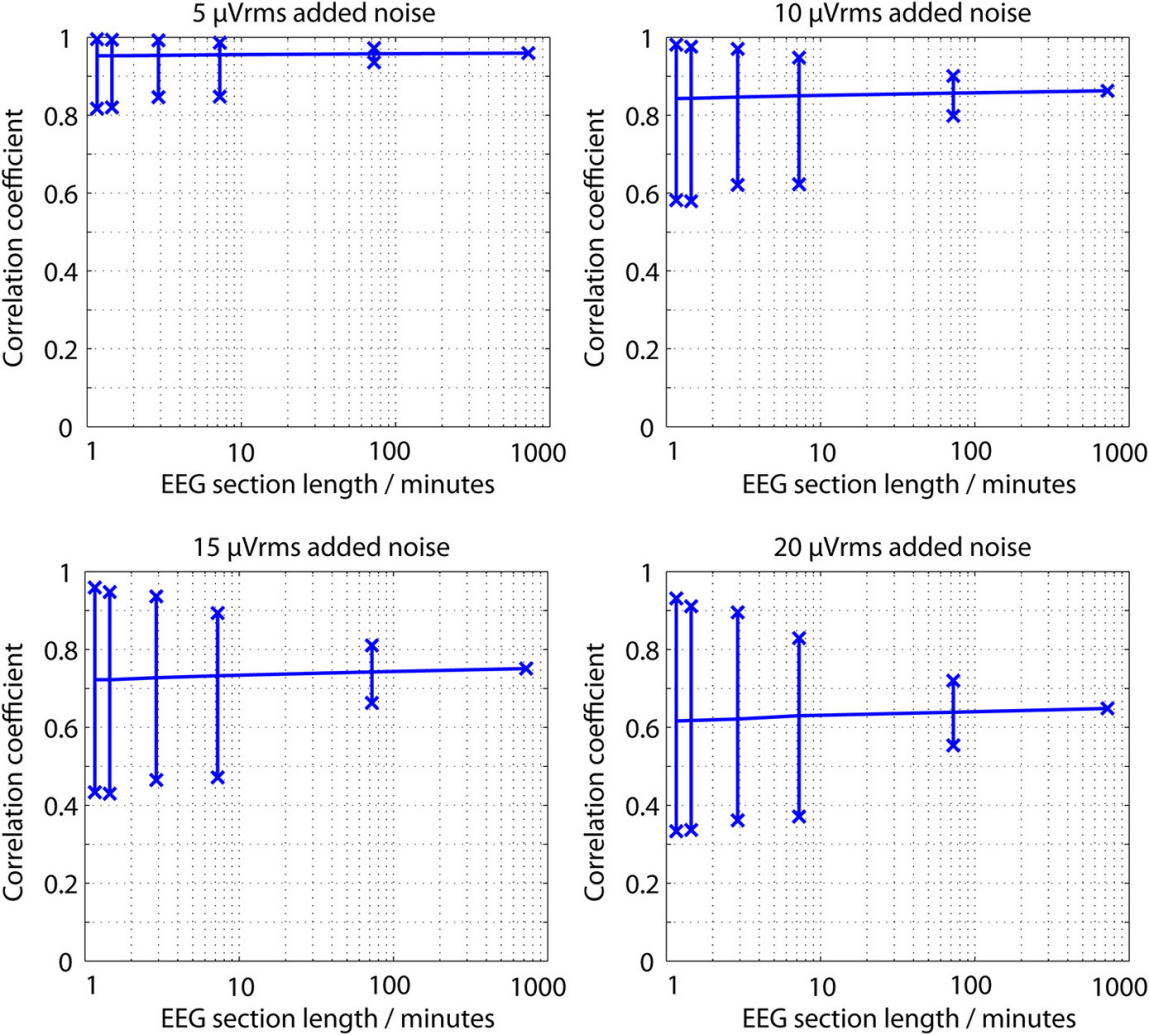
**Correlation coefficients between a raw EEG trace and a noise corrupted copy of the same EEG trace as the EEG section length used for calculation is changed**. Vertical lines show the maximum, minimum and median correlation values found over a complete 12.5 h EEG recording.

To put this noise level in context, Figure 6 shows the correlation coefficients between the 12.5 h raw recorded EEG trace and noise corrupted versions of it, as the coefficient is calculated over different time spans. Coefficients in excess of 0.9 are readily achieved, even in the presence of up to 15 μVrms noise. Partly this is because the underlying correlation present is not accurately estimated when very short sections of data are analyzed. There is a consistent tendency for the median correlation to be underestimated at the cost of much larger variances.

**Figure 7:**
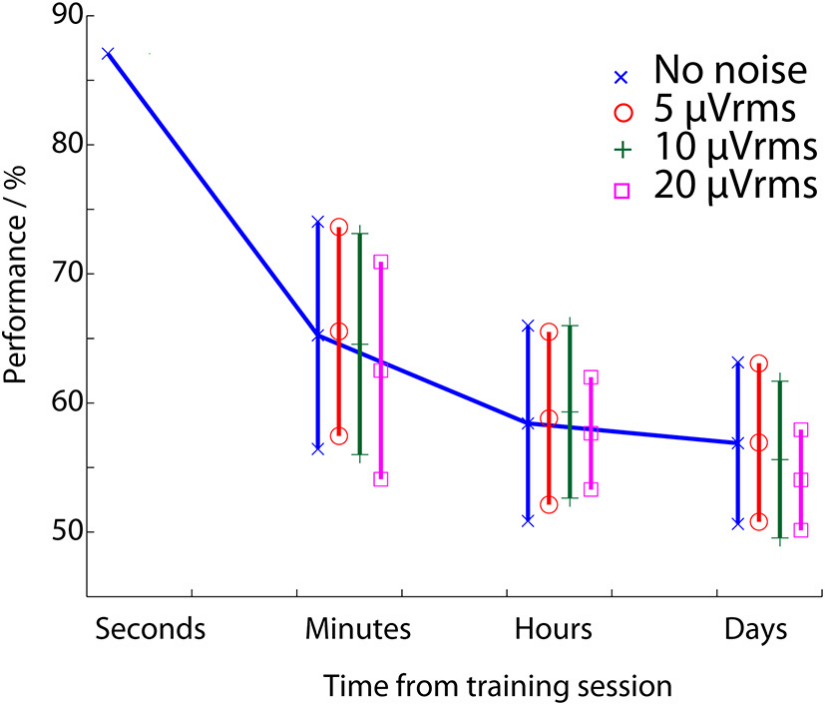
**Workload classification performance as a function of the time between the training and testing data set**. This is evaluated using the array of Artificial Neural Networks and injecting different levels of artificially generated noise into the input EEG signals.

Given this, the array of ANNs was tested using noise levels of 0, 5, 10, and 20 μVrms. The resulting temporal performance is summarized in Figure 7 which demonstrates the average classification performance of the *cross session* ANNs when all of the possible training/test configurations are used to investigate each time gap. In the no noise case this finds a very similar performance degradation to that in (Christensen et al., 2012, Figure 6). The achieved performance stabilizes over the time frame of hours to 56%, substantially below the starting performance level (86%), with the temporally independent cross validation results (73%) being between the two.

Christensen et al. (2012) demonstrated that some of this performance loss could be recovered by re-training the network with a small amount of known workload state data from each new EEG session, but this is undesirable due to the time and effort required. To avoid this process Figure 7 also tests the hypothesis of using noise-enhanced processing as a potential mitigation approach which does not require network re-training. A single noise case run of the parallel ANN configuration is presented in Figure 7. Apparent is that the 5 and 10 μVrms cases both show small improvements in classification performance over the time span of minutes to hours. Further, all of the noise levels show a reduction in the mean deviation of the performances, showing that the performance is less variable if additional noise is introduced. However, neither of these effects is substantial: the performance improvement in Figure 7 is approximately 0.3%, and the improvement in the results spread is less than 1% in most cases.

A repeated measures ANOVA, with null hypothesis that there is no difference between the mean performances in the noise and no noise cases, rejects the null hypothesis (*p* = 0.13 for the 5 μVrms, minutes case). This indicates that the mean performance change is not significant. Nevertheless, to demonstrate that the noise-enhancement in Figure 7 (which used a single run of the parallel ANN) is repeatable, 10 independent runs of the parallel ANN configuration have been performed with different input noise signals generated for each run. The performance values in the 5 μVrms of added noise case, when the training and test EEG data sections are minutes apart, are given in Table 2. This shows that no performance decreases are present. Instead there is a clear and consistent effect of improved performance with the small amount of noise added. Modeling the probability of the noise-enhanced algorithm being better than the no noise one as a Binomial distribution *B*(10, 0.5) (that is, there is a 50% chance of the noise added case outperforming the no noise case) the probability of all 10 noise added runs having better performance than the no noise one is less than 0.001. Assuming the noise added case is always better, *B*(10, 1), for the 10 runs performed the 95% confidence intervals go down to *p* = 0.69 suggesting as a lower bound that the performance is enhanced in approximately 70% of noise cases.

**Table 2:** **Classification performance when training and test data are taken from EEG sessions performed on the same day, minutes apart. Results are for 10 independent runs with 5 μVrms of added white Gaussian noise**.

**No noise**	**65.2%**
Noise run 1	65.6
Noise run 2	65.5
Noise run 3	65.6
Noise run 4	65.4
Noise run 5	65.7
Noise run 6	65.4
Noise run 7	65.5
Noise run 8	65.2
Noise run 9	65.3
Noise run 10	65.3

## 4. Discussion

This paper has presented a passive BCI based workload classification system investigating two of the most important factors for practical out-of-the-lab systems: the performance variation over time; and the noise robustness of the classification process. In-line with previous work, very good classification performance is achieved using little training data when the time gap between the training data and testing data is small.

### 4.1. Average and temporal performance

The cross validation ANNs, which used all of the possible training data regardless of when it was recorded compared to the test data, achieved an average performance level of 73%. This procedure is a standard approach for obtaining the best generalization performance when applied to new EEG data (maximizing criteria 1 from the introduction). However, it uses large amounts of test data from many different recordings to ensure the training data is as representative as possible. This is because when the training features are all temporally close together there is the potential for specific features, such as eye blinks or changes in muscle tone, to be present in the training sections which are not present in subsequent EEG recording sessions. This affects how Independent, Identically Distributed (IID) the feature vectors are, and places a lot of weight on a single training session to be representative in terms of equipment set up, user familiarity and user neurophysiology. Changes in performance in subsequent test sessions could then be due to a change in the mental status, or due to changes in the feature IID distributions.

It is for this reason that the *same session* tests, using ANNs trained using just 250 s of data (maximizing condition 2 from the introduction), achieved better performance (86%), but only over the short time frame where the EEG, feature IID distribution, and user mental state in the test period are very similar to that in the training period. As the time gap between the training data and the testing data increased there was a consistent decrease in the classification performance (Figure 4) as the used training EEG becomes less representative of the current testing EEG. This decrease in performance is apparent even over the relatively small time scale of minutes. Over larger time scales, substantial decreases in classification performance are observed with, as would be expected, the performance becoming worse than the 73% achieved when using more temporally spread training data. The average performance leveled off at approximately 57% for prolonged multi-day testing (Figure 7) with this degradation occurring over the time frame of hours. This performance level is above chance, but is unlikely to be meaningful for practical use. Moreover, a substantial variance in day-to-day performance was observed (Figure 5). On some days the existing neural network could be directly re-applied without further training of the network and very acceptable performances obtained. However, on many days the network achieved poor classification and would not be re-usable.

Only one point in the trade-off between the amount/spread of training data used and the amount/speed of the performance drop off has been investigated in this paper. Classically this trade-off is altered by using more training data: either in advance as in the cross validation approach; or by periodically re-training the network (periodically putting the user in a known workload state to generate new training data). Christensen et al. (2012) estimated that adding in 2.5 min of known workload state data per class was sufficient for re-training. However, generating this data requires time and effort, which places an emphasis on maximizing the time before re-training is required (maximizing condition 3 from the introduction).

### 4.2. Noise-enhanced processing

Noise-enhanced signal processing is proposed here as an algorithmic method for recovering some of the ANN performance when there is a large time gap between the training and test data sections, and so increasing the time before re-training is required. It has the benefits of not requiring any additional training data to be collected and being transparent to the end user.

*Stochastic resonance* is a well known but counter-intuitive effect where the performance of a non-linear system actually increases as more noise is present in the system (McDonnell and Abbott, 2009; McDonnell and Ward, 2011). Noise-resonance is commonly seen in biological systems, including neurons and the brain itself (Wiesenfeld and Moss, 1995; Gluckman, 1996; McDonnell and Abbott, 2009) and can be used in signal detection (Kay, 2000; Chen et al., 2007). For example the technique has been used for improving the performance of algorithms for detecting micro-calcifications (a key early sign of cancer) in breast mammograms (Peng et al., 2009), in radar target classification (Jouny, 2010), and is widely used in chromatography (Zhang et al., 2014). It has recently been applied to detecting transient signals in the EEG, both evoked and natural (Casson and Rodriguez-Villegas, 2011; Sampanna and Mitaim, 2013). Given the natural associations between noise and the EEG it is a very relevant technique to attempt to utilize, although it has not previously been applied to free-running EEG, suitable for passive BCI applications.

By using an array of ANNs to perform *testing with noise*, as a complement to the more common *training with noise*, Figure 5 shows that the workload classification process over the time span of days is robust to 5 and 10 μVrms of noise being added into the EEG traces. Figure 7 and Table 2 then demonstrate small stochastic resonance effects whereby the classification performance over the time span of minutes was consistently enhanced by the presence of small amounts of noise. To this end, the application of noise-enhancement has been successful, and this paper is the first demonstration of these effects in free-running EEG. However, while consistent, the achieved improvements are less than 1% and far too small to meaningfully eliminate any re-training required to get good multi-day use of the workload classification training with 250 s of data. It is clear that for free-running EEG (unlike evoked and transient EEG, Casson and Rodriguez-Villegas, 2011; Sampanna and Mitaim, 2013) the potential stochastic resonance effects are either very small or are not yet being exploited optimally.

Only one arrangement for introducing noise has been investigated here, but the process introduces many new degrees of freedom in terms of how much noise is added, its spectral composition and the parallel processing and multiple-runs options that it enables. Noise-enhancement and stochastic resonance effects are only just starting to be exploited in BCI applications, and if further established these new degrees of freedom are potentially highly interesting for re-visiting in other BCI problems where they can be exploited to improve performance.

## 5. Conclusions

This paper has investigated the temporal performance of Artificial Neural Networks for operator workload classification. Networks trained using 150 min of EEG data in a leave-one-out cross validation procedure obtained an average classification performance of 73% (Table 1). In contrast networks trained on only 250 s of EEG data, and so being much quicker to set up, achieved 86% average performance (Figure 4) over a short time frame when the test EEG/user state was very similar to the training data. However, these networks do not generalize well (due to changes in the IID distribution of the features, changes in user state, and similar) which leads to a drop off in classification performance as the time gap between the training and testing data increases (Figure 7). This shows that short training periods can be used with the ANN classifiers, but the classifiers will only work for a short amount of time.

To overcome this, noise-enhanced processing was explored as an algorithmic technique for recovering some of the lost performance, increasing the amount of time that the classifier can work for. Noise-enhanced processing is explored because it is potentially transparent to the end user and has interesting correlates with the design of EEG units/electrodes. The results (Table 2) show that noise enhanced processing has an effect: consistently better performances are obtained; although the current improvements are very small. Nevertheless, the consistent improvement is an interesting result and the first evidence that stochastic resonance effects could be exploited in free-running EEG and passive BCI applications.

## Funding

This work was carried out while the author was at Imperial College London and has been supported by the Junior Research Fellowship of Imperial College London and the Imperial College Open Access fund.

### Conflict of interest statement

The author declares that the research was conducted in the absence of any commercial or financial relationships that could be construed as a potential conflict of interest.

## Footnotes

^1^Competition contact for data: Justin Estepp, Air Force Research Laboratory, cognitive.state@gmail.com

## References

[B1] Ayaz H. Shewokis P. A. Bunce S. Izzetoglu K. Willems B. Onaral B. (2012). Optical brain monitoring for operator training and mental workload assessment. Neuroergonomics 59, 36–47. 10.1016/j.neuroimage.2011.06.023 21722738

[B2] Casson A. J. (2013). Towards noise-enhanced augmented cognition, in Foundations of Augmented Cognition, Vol. 8027, of Lecture Notes in Computer Science, eds Schmorrow D. D. Fidopiastis C. M. (Berlin; Heidelberg: Springer), 259–268 10.1016/j.jneumeth.2011.07.007

[B3] Casson A. J. Rodriguez-Villegas E. (2011). Utilising noise to improve an interictal spike detector. J. Neurosci. Methods 201, 262–268 10.1016/j.jneumeth.2011.07.007 21835203

[B4] Chen H. Varshney P. K. Kay S. M. Michels J. H. (2007). Theory of the stochastic resonance effect in signal detection: part I-fixed detectors. IEEE Trans. Signal Process. 55, 3172–3184 10.1109/TSP.2007.893757

[B5] Chi Y. Jung T.-P. Cauwenberghs G. (2010). Dry-contact and noncontact biopotential electrodes: methodological review. IEEE Rev. Biomed. Eng. 3, 106–119. 10.1109/RBME.2010.2084078 22275204

[B6] Christensen J. C. Estepp J. R. Wilson G. F. Russell C. A. (2012). The effects of day-to-day variability of physiological data on operator functional state classification. Neuroimage 59, 57–63. 10.1016/j.neuroimage.2011.07.091 21840403

[B7] Comstock J. R. Arnegard R. J. (1992). The Multi-Attribute Task Battery for Human Operator Workload and Strategic Behavior Research. Technical report, TM-104174, National Aeronautics and Space Administration Langley Research Center, Hampton, VA.

[B8] Cuadras A. Casas O. Pallas-Areny R. (2012). Power-noise trade-off in signal amplifiers, in IEEE I2MTC (Montevideo).

[B9] De Clercq W. Vergult A. Vanrumste B. Van Paesschen W. Van Huffel S. (2006). Canonical correlation analysis applied to remove muscle artifacts from the electroencephalogram. IEEE Trans. Biomed. Eng. 53, 2583–2587. 10.1109/TBME.2006.879459 17153216

[B10] Dijksterhuis C. de Waard D. Brookhuis K. Mulder B. de Jong R. (2013). Classifying visuomotor workload in a driving simulator using subject specific spatial brain patterns. Front. Neurosci. 7:149. 10.3389/fnins.2013.00149 23970851 PMC3748749

[B11] Duda R. O. Hart P. E. Stork D. G. (2001). Pattern Classification. New York, NY: Wiley.

[B12] Estepp J. R. Christensen J. C. Monnin J. W. Davis I. M. Wilson G. F. (2009). Validation of a dry electrode system for EEG, in Proceedings HFES (San Antonio, TX).

[B13] Estepp J. R. Klosterman S. L. Christensen J. C. (2011). An assessment of non-stationarity in physiological cognitive state assessment using artificial neural networks, in IEEE EMBC (Boston, MA). 10.1109/IEMBS.2011.6091616 22255840

[B14] Gandhi N. Khe C. Chung D. Chi Y. M. Cauwenberghs G. (2011). Properties of dry and non-contact electrodes for wearable physiological sensors, in International Conference BSN (Dallas, TX).

[B15] Gargiulo G. Bifulco P. Calvo R. A. Cesarelli M. Jin C. van Schaik A. (2008). A mobile EEG system with dry electrodes, in IEEE BioCAS (Baltimore, MD).

[B16] Gluckman B. J. (1996). Stochastic resonance in a neuronal network from mammalian brain. Phys. Rev. Lett. 77, 4098–4101. 10.1103/PhysRevLett.77.4098 10062387

[B17] Harrison R. R. Charles C. (2003). A low-power low-noise CMOS amplifier for neural recording applications. IEEE J. Solid State Circuits. 38, 958–965 10.1109/JSSC.2003.811979

[B18] Huigen E. Peper A. Grimbergen C. A. (2002). Investigation into the origin of the noise of surface electrodes. Med. Biol. Eng. Comput. 40, 332–338. 10.1007/BF02344216 12195981

[B19] IMEC (2012). Holst Centre and Panasonic Present Wireless Low-Power Active-Electrode EEG Headset. Available online at: http://www.imec.be/

[B20] Jouny I. (2010). Stochastic resonance and suboptimal radar target classification. Proc. SPIE 7696, 729612 10.1117/12.852552

[B21] Kay S. (2000). Can detectability be improved by adding noise? IEEE Signal Process. Lett. 7, 8–10 10.1109/97.809511

[B22] Matthews R. McDonald N. J. Hervieux P. Turner P. J. Steindorf M. A. (2007). A wearable physiological sensor suite for unobtrusive monitoring of physiological and cognitive state, in IEEE EMBC (Lyon). 10.1109/IEMBS.2007.4353532 18003198

[B23] McDonnell M. D. Abbott D. (2009). What is stochastic resonance? definitions, misconceptions, debates, and its relevance to biology. PLoS Comput. Biol. 5:e1000348. 10.1371/journal.pcbi.1000348 19562010 PMC2660436

[B24] McDonnell M. D. Ward L. M. (2011). The benefits of noise in neural systems: bridging theory and experiment. Nat. Rev. Neurosci. 12, 415–426. 10.1038/nrn3061 21685932

[B25] Miller W. D. Jr. (2010). The U.S. Air Force-Developed Adaptation of the Multi-Attribute Task Battery for the Assessment of Human Operator Workload and Strategic Behavior, Technical report, AFRL-RH-WP-TR-2010-0133, U.S. Air Force Research Laboratory, Wright-Patterson airforce base.

[B26] Patki S. Grundlehner B. Verwegen A. Mitra S. Xu J. Matsumoto A. (2012). Wireless EEG system with real time impedance monitoring and active electrodes, in IEEE BioCAS (Hsinchu).

[B27] Peng R. Chen H. Varshney P. (2009). Noise-enhanced detection of micro-calcifications in digital mammograms. IEEE J. Sel. Areas Signal Process. 3, 62–73 10.1109/JSTSP.2008.2011162

[B28] Rechtschaffen A. Kales A. (eds.). (1968). A Manual of Standardized Terminology, Techniques and Scoring System for Sleep Stages of Human Subjects. Washington, DC: Public Health Service, U.S. Government Printing Office.

[B29] Sampanna R. Mitaim S. (2013). Noise enhanced array signal detection in P300 speller paradigm using ICA-based subspace projections, in IEEE EMBC (Osaka). 10.1109/EMBC.2013.6610481 24110668

[B30] Schmorrow D. D. Stanney K. M. (eds.). (2008). Augmented Cognition: A Practitioner's Guide. Santa Monica, CA: Human Factors and Ergonomics Society.

[B31] Slater J. D. Kalamangalam G. P. Hope O. (2012). Quality assessment of electroencephalography obtained from a “dry electrode” system. J. Neurosci. Methods 208, 134–137. 10.1016/j.jneumeth.2012.05.011 22633894

[B32] Vergult A. De Clercq Q. Palmini A. Vanrumste B. Dupont P. Van Huffel S.. (2007). Improving the interpretation of ictal scalp EEG: BSS-CCA algorithm for muscle artifact removal. Epilepsia 45, 950–958. 10.1111/j.1528-1167.2007.01031.x 17381439

[B33] Wiesenfeld K. Moss F. (1995). Stochastic resonance and the benefits of noise: from ice ages to crayfish and SQUIDs. Nature 373, 33–36. 10.1038/373033a0 7800036

[B34] Wilson G. F. Russell C. A. (2007). Performance enhancement in a UAV task using psychophysiological determined adaptive aiding. Hum. Factors 49, 1005–1019. 10.1518/001872007X249875 18074700

[B35] Xu J. Yazicioglu R. F. Grundlehner B. Harpe P. Makinwa K. A. A. Van Hoof C. (2011). A 160 μW 8-channel active electrode system for EEG monitoring. IEEE Trans. Biomed. Circuits Syst. 5, 555–567. 10.1109/TBCAS.2011.2170985 23852553

[B36] Zander T. O. Kothe C. (2011). Towards passive braincomputer interfaces: applying braincomputer interface technology to humanmachine systems in general. J. Neural Eng. 8:025005. 10.1088/1741-2560/8/2/025005 21436512

[B37] Zhang W. Guo J. Xiang B. Fan H. Xu F. (2014). Improving the detection sensitivity of chromatography by stochastic resonance. Analyst 139, 2099–2107. 10.1039/C3AN02192K 24622614

